# A novel prognostic model based on urea cycle-related gene signature for colorectal cancer

**DOI:** 10.3389/fsurg.2022.1027655

**Published:** 2022-10-21

**Authors:** Haiyang Guo, Yuanbiao Wang, Lei Gou, Xiaobo Wang, Yong Tang, Xianfei Wang

**Affiliations:** ^1^ Department of Gastroenterology, Affiliated Hospital of North Sichuan Medical College, Nanchong, China; ^2^ Department of Yunnan Tumor Research Institute, The Third Affiliated Hospital of Kunming Medical University, Yunnan Cancer Hospital, Kunming, China

**Keywords:** colorectal cancer, urea cycle, Gene signature, prognosis, immune infiltration

## Abstract

**Background:**

Colorectal cancer (CRC) is the second leading cause of cancer-related deaths in the world. This study aimed to develop a urea cycle (UC)-related gene signature that provides a theoretical foundation for the prognosis and treatment of patients with CRC.

**Methods:**

Differentially expressed UC-related genes in CRC were confirmed using differential analysis and Venn diagrams. Univariate Cox and least absolute shrinkage and selection operator regression analyses were performed to identify UC-related prognostic genes. A UC-related signature was created and confirmed using distinct datasets. Independent prognostic predictors were authenticated using Cox analysis. The Cell-type Identification by Estimating Relative Subsets of RNA Transcripts algorithm and Spearman method were applied to probe the linkage between UC-related prognostic genes and tumor immune-infiltrating cells. The Human Protein Atlas database was used to determine the protein expression levels of prognostic genes in CRC and normal tissues. Verification of the expression levels of UC-related prognostic genes in clinical tissue samples was performed using *real-time quantitative polymerase chain reaction (qPCR)*.

**Results:**

A total of 49 DEUCRGs in CRC were mined. Eight prognostic genes (TIMP1, FABP4, MMP3, MMP1, CD177, CA2, S100P, and SPP1) were identified to construct a UC-related gene signature. The signature was then affirmed using an external validation set. The risk score was demonstrated to be a credible independent prognostic predictor using Cox regression analysis. Functional enrichment analysis revealed that focal adhesion, ECM-receptor interaction, IL-17 signaling pathway, and nitrogen metabolism were associated with the UC-related gene signature. Immune infiltration and correlation analyses revealed a significant correlation between UC-related prognostic genes and differential immune cells between the two risk subgroups. Finally, the qPCR results of clinical samples further confirmed the results of the public database.

**Conclusion:**

Taken together, this study authenticated UC-related prognostic genes and developed a gene signature for the prognosis of CRC, which will be of great significance in the identification of prognostic molecular biomarkers, clinical prognosis prediction, and development of treatment strategies for patients with CRC.

## Introduction

According to global cancer data, colorectal cancer (CRC) ranks third in incidence and second in mortality rate worldwide. GLOBOCAN estimated more than 1.9 million new CRC cases and 935,000 deaths in 2020 (1, 2). The prognosis and survival status of patients with CRC have not improved significantly over the years (3). The prognosis of CRC is predicted using the tumor–node–metastasis (TNM) staging system, histopathologic criteria, and tumor markers, but it cannot accurately predict the clinical prognosis (4). The etiology of CRC is complex, with approximately 10% patients having susceptibility to germline mutations that lead to familial cases. However, the majority of patients with CRC have sporadic cancer caused by a combination of environmental and genetic risk factors (5), although the specific molecular mechanisms remain unknown. Therefore, the molecular mechanisms underlying the development of CRC should be explored further, and novel biomarkers for prognostic assessment should be identified to improve the clinical prognosis of patients. It is challenging to predict the prognosis of CRC owing to its rapid progression and highly heterogeneous nature (6). This necessitates the development of new prognostic models.

The process by which ammonia produced during the metabolism of amino acids in the body is converted into urea *via* ornithine constitutes the urea cycle (UC). UC eliminates the excess nitrogen and ammonia produced by the breakdown of proteins or the synthesis of nitrogenous compounds in the body. UC enzymes also manipulate nucleotide metabolism in certain types of tumors. UC-related genes (UCRGs) are overexpressed or silenced in different types of cancers, and altered UC gene expression is actively involved in tumorigenesis (7). UC dysregulation (UCD) is observed in various cancer types and is associated with a poor prognosis, but is responsive to immunotherapy (8). Tumor cells reprogram their metabolism to maximize the use of nitrogen and carbon to obtain sufficient energy for tumor proliferation and rapid growth (9, 10). p53 inhibits tumor growth by regulating ammonia metabolism *via* UC, thereby controlling polyamine synthesis in tumor cells (11). UC enzymes play a critical role in killing cancer cells and suppressing cancer growth. In the human hepatoblastoma cell line, HepG2, deficiency of arginase 1 and ornithine carbamoyltransferase (OTC) results in high ammonia levels and diminished UC function (12). OTC and argininosuccinate synthase (ASS) levels are deficient in acute lymphoblastic leukemia (13). The UC enzyme, carbamoyl-phosphate synthase 1 (CPS1), maintains the pyrimidine pool in non-small cell lung cancer by activating CAD, and silencing CPS1 in KL cells induces cell death and inhibits tumor growth *in vivo* due to the depletion of pyrimidines (14). ASS1 expression promotes CRC cell proliferation and tumor formation *in vitro* (15). Whether UCRGs are associated with the prognosis of patients with CRC remains unclear; therefore, systematic studies of prognosis-related UC genes can help to understand their role in CRC development and progression and aid in guiding clinical decisions.

This study aimed to determine the prognostic value of UCRGs in patients with CRC. RNA-sequencing (seq) data for CRC and the corresponding clinical data were extracted from public databases. UC-associated differentially expressed genes (DEGs) closely associated with prognosis were identified to construct a predictive model for CRC prognosis in The Cancer Genome Atlas (TCGA) cohort. We then validated it in the Gene Expression Omnibus (GEO) cohort, determined the expression levels of eight genes in colon cancer tissues using quantitative reverse transcription-polymerase chain reaction (qRT-PCR), and obtained results consistent with our initial prediction.

## Methods

### Gene and dataset collection

We integrated the transcriptomic data of 673 CRC and 51 normal tissue samples using TCGA database (https://portal.gdc.cancer.gov/repository). Seven CRC datasets were mined from the GEO database (https://www.ncbi.nlm.nih.gov/geo/) (GSE41258, GSE110223, GSE110224, GSE113513, GSE17538, GSE39582, and GSE44076) and used in this study. The number of tissue samples and their usage in each dataset are listed in Table 1. A total of 2,857 UCRGs were derived from the GeneCard database (https://www.genecards.org/) by searching “UC” and are listed in Supplementary Table S1. A flow chart of the present study is shown in Supplementary Figure S1.

**Table 1:** Sample size and usage of the datasets.

Dataset	Normal samples	CRC samples	Usage
TCGA-CRC	51	622	DEGs analysis and training set for prognosis
GSE41258	54	185	DEGs analysis
GSE110223	13	13	DEGs analysis
GSE110224	17	17	DEGs analysis
GSE113513	14	14	DEGs analysis
GSE17538		232	Validation set for prognosis
GSE39582	19	566	Validation of gene expression
GSE44076	50	98	Validation of gene expression

### Identification of differentially expressed UCRGs (DEUCRGs) in CRC

On the basis of *p* value < 0.05 and |log_2_FoldChange(FC)| > 1, we first identified the DEGs (CRC samples *vs.* normal samples) in the TCGA-CRC, GSE41258, GSE110223, GSE110224, and GSE113513 datasets using the limma package (16). We separately crossed the upregulated and downregulated genes in the five datasets to identify the shared DEGs, which were further intersected with UCRGs to identify DEUCRGs for subsequent prognostic analysis.

### Functional annotation analysis

We used the R package clusterProfiler (17) for Gene Ontology (GO) and Kyoto Encyclopedia of Genes and Genomes (KEGG) enrichment analyses. GO was categorized as cellular component (CC), molecular function (MF), and biological process (BP). The significance criterion was adjusted *p* value ≤ 0.05.

### Establishment of UC-related gene signature in CRC

The 590 patients with CRC with survival information in TCGA-CRC dataset served as the training set and 232 patients with survival information from the GSE17538 dataset were used as the external validation set to create and verify the UC-related gene signature for predicting the survival of patients with CRC. Univariate Cox and least absolute shrinkage and selection operator (LASSO) regression analyses were used to select the UC-related prognostic genes in the training set. Depending on the risk score formula (Riskscore = $\sum_{1}^{n}\mathrm{coef}(\mathrm{genei})*\exp\mathrm{ression}(\mathrm{genei})$, coef represents the coefficient obtained by LASSO), and cut-off value calculated using the surv_cutpoint function in the cutoff package, patients were classified into two risk subgroups: high- and low-risk groups. Kaplan–Meier curves, receiver operating characteristic (ROC) analysis, and risk curves were used to demonstrate the predictive efficiency of the gene signature.

### Relevance analysis of UC-related gene signature and clinical parameters

Risk scores for different clinical factor subgroups were compared using the Wilcoxon or Kruskal–Wallis test. Stratified survival analysis for different clinical subgroups was also performed by developing K–M curves.

### Independent prognostic analysis and nomogram creation

The risk score, age, sex, race, stage, pathologic_M, pathologic_N, and pathologic_T were included in Cox analyses (univariate Cox and multivariate Cox) to authenticate independent prognostic predictors. The nomogram comprising the independent prognostic predictors was drawn using R language “rms” to predict survival at 1, 3, and 5 years in patients with CRC. The corresponding calibration and DCA curves were plotted to appraise the precision and reliability of the nomogram model predictions.

### Relevance analysis of UC-related prognostic genes and immune infiltration

Discrepancies in 22 types of immune infiltrating cells were assessed and compared between the two risk groups using the Cell-type Identification by Estimating Relative Subsets of RNA Transcripts (CIBERSORT) algorithm (18) and Wilcoxon test. We calculated the correlation between UC-related prognostic genes and differential immune cells using the Spearman's method.

### Analysis and validation of the expression levels of UC-related prognostic genes

We first verified the discrepancy in the expression levels of UC-related prognostic genes in CRC and normal samples in the external datasets, GSE39582 and GSE44076. Corresponding box-line plots were obtained using the ggplot2 package. We used immunohistochemistry images from the Human Protein Atlas (HPA) database (https://www.proteinatlas.org/) to further determine the protein expression levels of UC-related prognostic genes in normal and CRC tissues. To further confirm the results of the public database analysis, we collected six normal tissue samples and six CRC tissue samples from the Affiliated Hospital of North Sichuan Medical College in June and performed RNA isolation and qRT-PCR. This study was approved by the Affiliated Hospital of North Sichuan Medical College (Nanchong, China) (Ethical Application Ref: 2022ER237-1). Total RNA from 12 samples was separated using TRIzol (Ambion, USA), according to the manufacturer's instructions. The inverse transcription of total RNA into cDNA was implemented using the First-strand cDNA synthesis kit (Servicebio, China), according to the manufacturer's instructions. Then, qPCR was carried out using the 2xUniversal Blue SYBR Green qPCR Master Mix (Servicebio, China), according to the manufacturer's instructions. The primer sequences used for PCR are listed in Supplementary Table S2. Expression was normalized to the internal reference glyceraldehyde 3-phosphate dehydrogenase and computed using the 2−*ΔΔ*Cq method (19).

### Statistical analysis

All bioinformatics analyses were performed using the R language. Wilcoxon test or Kruskal–Wallis test was used to compare data from different groups. Student's *t*-test was used to compare the discrepancies in qRT-PCR.

## Results

### DEUCRGs in CRC

**Figure 1:**
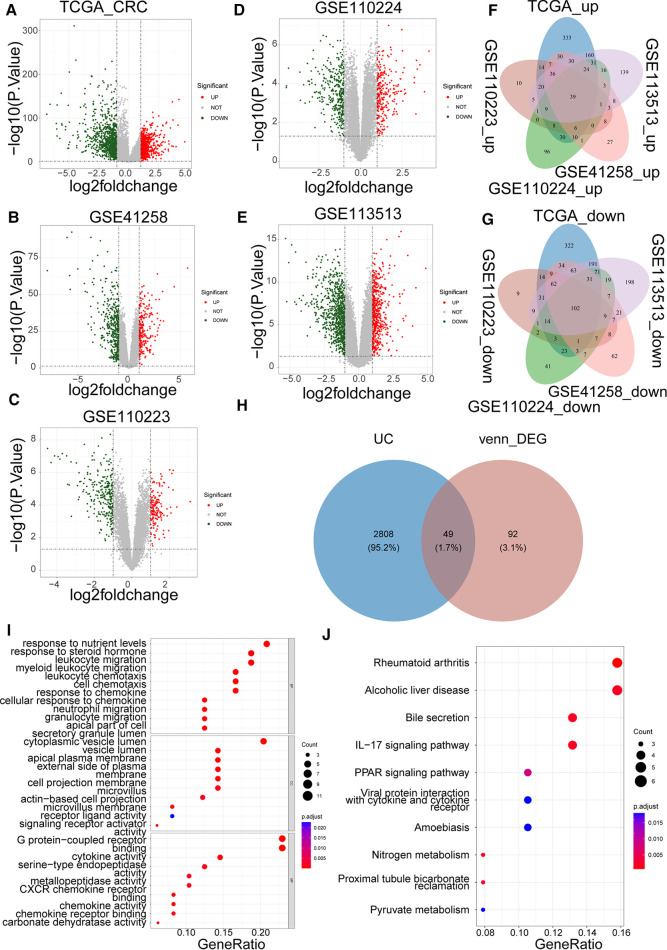
Identification of colorectal cancer (CRC)-associated differentially expressed UC-related genes (DEUCRGs) in the cancer genome atlas (TCGA) and gene expression omnibus (GEO) cohorts. Volcano plots of DEGs in (**A**) TCGA-CRC, (**B**) GSE110223, (**C**) GSE41258, (**D**) GSE110224, and (**E**) GSE113513 datasets. (**F**) Venn diagram of upregulated genes in five datasets. (**G**) Venn diagram of downregulated genes in five datasets. (**H**) Venn diagram for identifying DEUCRGs. (**I**) Top 10 Gene Ontology (GO) terms enriched by DEUCRGs under cellular component (CC), molecular function (MF), and biological process (BP) subcategories. (**J**) Top 10 Kyoto Encyclopedia of Genes and Genomes (KEGG) pathways enriched by DEUCRGs.

Based on |log_2_FC| > 1 and *p* value < 0.05, we first identified the DEGs (CRC samples *vs.* normal samples) in TCGA-CRC, GSE41258, GSE110223, GSE110224, and GSE113513 datasets. Volcano plots of DEGs for each dataset are shown separately in Figures 1A–E. The specific numbers of DEGs per dataset are presented in Table 2. We intersected the upregulated and downregulated genes in the CRC samples from each of the above five datasets, resulting in 39 crossed upregulated genes and 102 crossed downregulated genes (Figures 1F–G; Supplementary Table S3). We then intersected the 141 crossed differential genes mentioned above with the 2,857 UCRGs derived from the GeneCard database, resulting in 49 crossed genes, namely DEUCRGs in CRC (Figure 1H; Supplementary Table S3). To further probe the function of the DEUCRGs in CRC, functional enrichment analysis was performed. As shown in Supplementary Table S4, 149 GO items (92 BP, 20 CC, and 37 MF items) and 17 KEGG pathways were identified. The top 10 items under each classification are shown in the bubble diagram (Figures 1I–J). These genes were mainly linked to immune-, extracellular matrix-, hormone metabolism-, and nitrogen metabolism-related biological processes. Furthermore, these genes were implicated in “Bile secretion,” “Nitrogen metabolism,” “IL-17 signaling pathway,” “Proximal tubule bicarbonate reclamation,” “PPAR signaling pathway,” “Pyruvate metabolism,” “NF-kappa B signaling pathway,” and “Chemokine signaling pathway.”

**Table 2:** The number of DEGs in each dataset.

Dataset	DEGs	Up-regulated genes	Down-regulated genes
TCGA-CRC	1761	787	974
GSE41258	666	233	433
GSE110223	455	167	288
GSE110224	616	275	341
GSE113513	1361	525	836

### UC-related gene signature to assess the prognosis of patients with CRC

**Figure 2:**
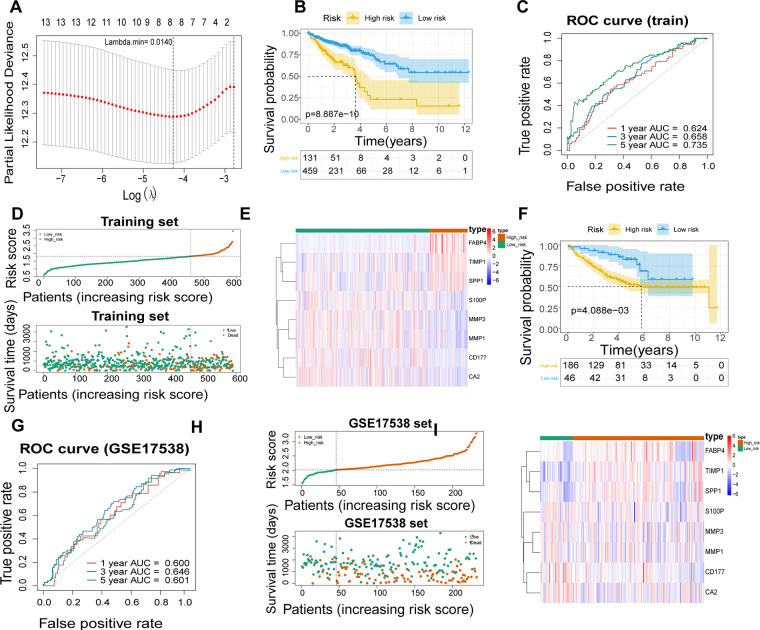
Establishment of a UC- relevant risk score model. (**A**) Least absolute shrinkage and selection operator (LASSO) regression analysis to establish an eight-gene signature. (**B**) Kaplan–Meier curves of the high- and low-risk groups in TCGA-CRC dataset. (**C**) Receiver operating characteristic (ROC) curves of 1, 3, and 5 years overall survival (OS) in TCGA-CRC dataset. (**D**) Signature-based distribution of risk scores and survival status in TCGA-CRC dataset. (**E**) Differences in prognostic gene expression in different risk groups in TCGA-CRC dataset. (**F**) Kaplan–Meier Curve of the high- and low-risk groups in GSE17538 dataset. (**G**) ROC curves of 1, 3, and 5 years OS in GSE17538 dataset. (**H**) Distribution of risk scores and survival status in GSE17538 dataset. (**I**) Differences in prognostic gene expression in different risk groups in GSE17538 dataset.

A total of 590 patients with CRC with survival information in TCGA-CRC dataset served as the training set. To mine the UCRGs relevant to the overall survival (OS) of patients with CRC, we incorporated the 49 DEUCRGs obtained above into a univariate Cox analysis in the training set. Fourteen genes associated with OS of patients with CRC were selected (*p* < 0.1), with tissue inhibitor of metalloproteinase-1 (TIMP1), fatty acid-binding protein 4 (FABP4), secreted phosphoprotein 1 (SPP1), and butyrylcholinesterase as risk factors for CRC prognosis [hazard ratio (HR) > 1], and matrix metallopeptidase (MMP)-3, MMP1, C-X-C motif chemokine ligand (CXCL)-1, CXCL3, CD177, carbonic anhydrase (CA)-2, S100 calcium-binding protein *P* (S100P), CA4, nuclear receptor subfamily 3 group C member 2, and CXCL8 as protective factors for CRC prognosis (HR < 1) (Table 3). The above fourteen genes were further integrated into LASSO analysis with a 20-fold cross-validation. As shown in Figure 2A, when lambda min was 0.0140, the corresponding number of genes was eight. Therefore, eight genes (TIMP1, FABP4, MMP3, MMP1, CD177, CA2, S100P, and SPP1) and their corresponding coefficients were determined as UC-related prognostic genes for signature establishment (Table 4). Next, we developed a risk signature based on the following formula: RiskScore = (–0.02030967)×expression (MMP3) + (–0.099075148)×expression (MMP1) + (–0.041645445)×expression(CD177) + (–0.019591304)×expression (CA2) + (–0.074156971)×expression (S100P) + 0.356587004×expression (TIMP1) + 0.06708291×expression (FABP4) + 0.00844555 × expression (SPP1). Based on this formula, we calculated the risk score for each patient with CRC in the training set and classified them into high- and low-risk groups based on the cutoff value. The K–M curve illustrated that patients with higher risk had significantly poorer survival than those with lower risk (Figure 2B). We plotted the ROC curves to check the predictive efficiency of the signature. The area under the curve values for OS in the training set were 0.624 (1 year), 0.658 (3 years), and 0.735 (5 years), indicating decent accuracy (Figure 2C), the cut-off values of the ROC curves were shown in Supplementary Figures S2A–C. Figure 2D shows the distribution of the ranked risk score and survival status for each patient in the training set. Survival status showed that, as the risk score increased, patients had a relatively high risk of death. The expression heatmap showed that FABP4, TIMP1, and SPP1 were highly expressed in patients with high risk scores, whereas S100P, MMP3, MMP1, CD177, and CA2 were highly expressed in patients with low risk scores (Figure 2E). To further prove the applicability and reliability of the risk signature, the above analysis was carried out in the external validation set (GSE17538 dataset). A comparable trend was observed in the external validation set (Figures 2F–I), the cut-off values of the ROC curves were shown in Supplementary Figures S2D–F. The above results confirmed that the UC-related gene signature is a valid survival predictor for patients with CRC.

**Table 3:** The genes identified by univariate Cox.

Gene ID	HR	HR.95L	HR.95H	*p* value
TIMP1	1.451755314	1.161333836	1.814804172	0.001062858
FABP4	1.187141941	1.059480001	1.330186494	0.003123349
MMP3	0.8747019	0.793471449	0.964248197	0.007100919
MMP1	0.890853127	0.815415718	0.973269556	0.010463281
CXCL1	0.850727309	0.750220785	0.964698617	0.011727482
CXCL3	0.848093355	0.733223093	0.980959747	0.026497115
CD177	0.874464632	0.768124005	0.995527268	0.042588229
CA2	0.896063276	0.80535475	0.996988464	0.043867832
S100P	0.876935588	0.769974692	0.998754937	0.047845726
SPP1	1.083364054	0.997208995	1.176962583	0.05824383
CA4	0.901704615	0.808107267	1.006142681	0.064249679
NR3C2	0.806037218	0.635387808	1.022518828	0.075651532
BCHE	1.337315354	0.963650024	1.855873307	0.082122733
CXCL8	0.915782801	0.827164463	1.013895274	0.090224387

**Table 4:** The gene coefficients obtained from LASSO analysis.

Gene	Coef
TIMP1	0.356587004
FABP4	0.06708291
MMP3	−0.02030967
MMP1	−0.099075148
CD177	−0.041645445
CA2	−0.019591304
S100P	−0.074156971
SPP1	0.00844555

### Relevance analysis of clinical parameters and UC-related gene signature

**Figure 3:**
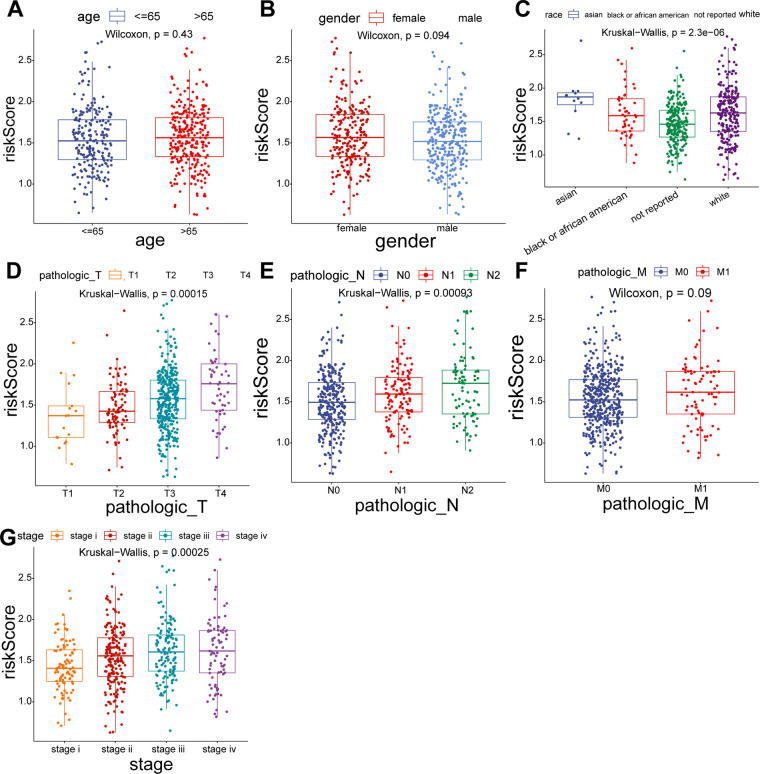
Comparison of risk scores for different subgroups of clinical parameters. (**A**) Age > 65 *vs.* Age ≤ 65. (**B**) Female *vs.* Male. (**C**) Asian *vs.* Black *vs.* White. (**D**) T1 *vs.* T2 *vs.* T3 *vs.* T4. (**E**) N0 *vs.* N1 *vs.* N2. (**F**) M0 *vs.* M1. (**G**) Stage I *vs.* II *vs.* III *vs.* IV.

**Figure 4:**
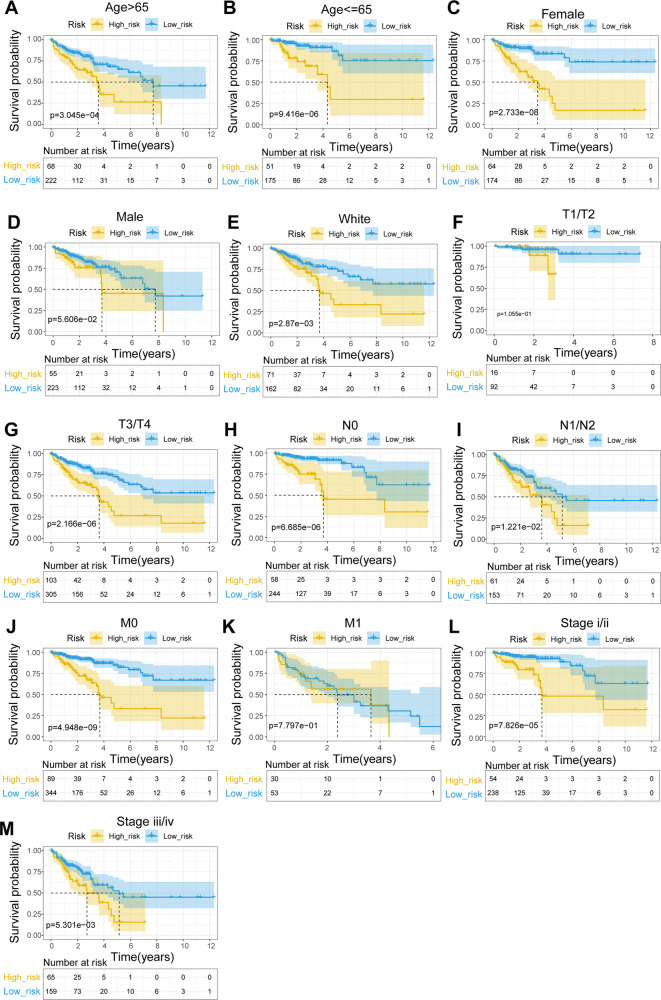
Stratified survival analysis of subgroups with different clinical parameters. (**A**) Age > 65. (**B**) Age ≤ 65. (**C**) Female. (**D**) Male. (**E**) White. (**F**) T1/T2. (**G**) T3/T4. (**H**) N0. (**I**) N1–N2. (**J**) M0. (**K**) M1. (**L**) Stage I/II. (**M**) Stage III/IV.

To explore the relationship between the UC-related gene signature and clinical factors, we compared the risk scores of different clinical subgroups of patients. As shown in Figures 3A–C, UC-related risk scores were independent of sex and age and correlated with race. Additionally, we found that the UC-related risk scores were notably correlated with stage, pathologic_M, pathologic_N, and pathologic_T stage, and the risk scores tended to increase as the malignancy of the tumor (Figures 3D–G). We further explored the application of the risk score in patients with different clinicopathological characteristics. The results of the stratified survival analysis uncovered significant differences in the survival of the two groups for most clinical subgroups, including age ≤ 65 years, age > 65 years, female sex, white ethnicity, M0, N0, N1/N2, T3/T4, stage I/II, and stage III/IV subgroups (Figure 4).

### UC-related gene signature is an independent prognostic predictor

**Figure 5:**
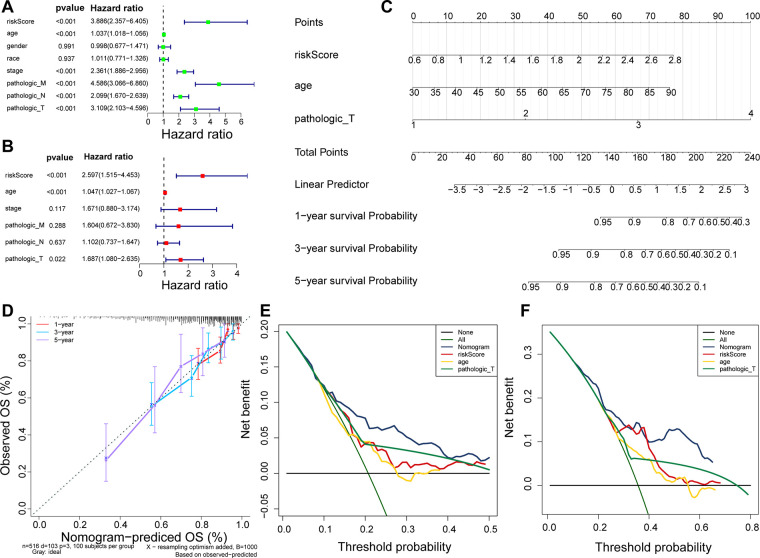
Nomogram predicting the OS of patients with CRC. (**A**) Univariate Cox regression analysis in TCGA cohort. (**B**) Multivariate Cox regression analysis in TCGA cohort. (**C**) Nomogram predicting patient survival at 1, 3, and 5 years. (**D**) Calibration curves of nomogram. (**E**) DCA curve for predicting 3-year survival of patients. (**F**) DCA curve for predicting 5-year survival of patients.

Using Cox analyses (univariate and multivariate Cox), we determined that risk score, age, and pathologic_T were independent prognosis predictors in patients with CRC (Figures 5A,B; *p* value < 0.05). A nomogram containing independent prognostic predictors was generated (Figure 5C), and the calibration curves proved that the performance of the nomogram in predicting the survival at 1, 3, and 5 years in patients with CRC was satisfactory (Figure 5D), whereas the DCA curves revealed that the nomogram had a higher accuracy in predicting the survival of patients at 3 and 5 years than the individual independent predictors (risk score, age, and T) (Figures 5E–F).

### Association of UC-related gene signature with immune infiltrating cells

**Figure 6:**
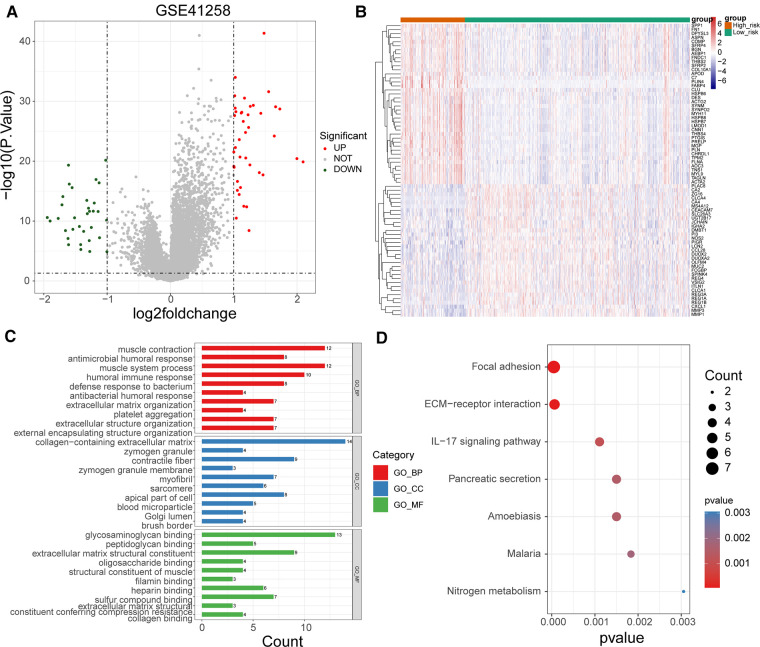
Functional enrichment of DEGs between high- and low-risk groups. (**A**) Volcano map of DEGs between high- and low-risk groups. (**B**) Heat map of DEGs between high- and low-risk groups (**C**) Top 10 GO terms enriched by DEGs between high- and low-risk groups under BP, CC, and MF subcategories. (**D**) KEGG pathways enriched by DEGs between high- and low-risk groups.

**Figure 7:**
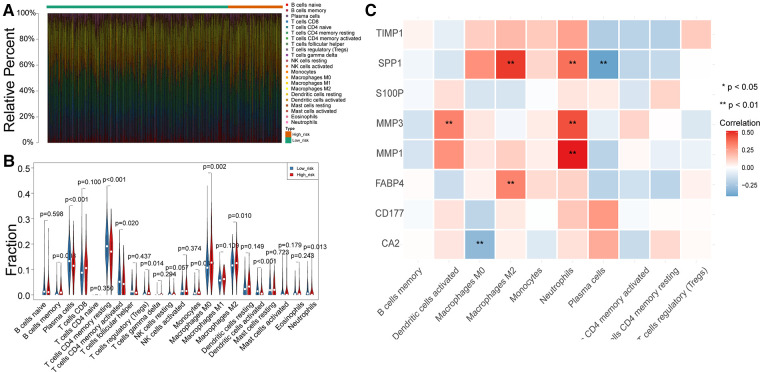
Differences in immune infiltrating cells between the high- and low-risk patients with CRC. (**A**) Rrelative percent of immune infiltrating cells in each CRC sample. (**B**) Violin plot of immune infiltrating cells between high- and low-risk groups. (**C**) Correlation between UC-related prognostic genes and differential immune infiltrating cells.

To uncover potential mechanisms for the disparity in the prognosis of the two risk groups, we performed the authentication and functional enrichment analysis of DEGs between the two groups. As shown in Figures 6A–B, a total of 73 DEGs (high-risk *vs.* low-risk), containing 40 highly expressed and 33 lowly expressed genes, were identified. Accordingly, 24 BP items, 36 CC items, 24 MF items, and seven KEGG pathways were identified (Supplementary Table S5). The top 10 GO items for each classification are shown in Figure 6C. We observed that the above genes were principally linked to the humoral immune response, antibacterial humoral response, and extracellular matrix organization. Furthermore, these genes were implicated in focal adhesion, ECM-receptor interaction, IL-17 signaling pathway, and nitrogen metabolism (Figure 6D). As immune-relevant biological processes and pathways were revealed to be connected to the UC-related gene signature, we investigated the changes in immune infiltration between the two risk groups. Using the CIBERSORT algorithm, 124 samples in the high-risk group and 430 samples in the low-risk group were incorporated to compute the fraction of each immune infiltration cell after excluding samples with *p* value > 0.05 (Figure 7A). As shown in the violin plot, the fraction of B cell memory, T-regulatory cells, monocytes, and macrophages M0 and M2 was elevated in patients with high risk scores, while the fraction of T cells CD4 memory resting, T cells CD4 memory activated, plasma cells, activated dendritic cells, and neutrophils was higher in patients with low risk scores (Figure 7B). We further calculated the relevance of the UC-related prognostic genes and the 10 differential immune cells mentioned above using the Spearman method (Supplementary Table S6). Based on the thresholds of |cor| > 0.3 and *p* < 0.05, we discovered that SPP1 was positively correlated with macrophage M2 and neutrophils and negatively correlated with plasma cells (Figure 7C). MMP3 expression was significantly and positively correlated with dendritic cell activation and neutrophils (Figure 7C). MMP1 expression was significantly positively correlated with neutrophil levels (Figure 7C). FABP4 was positively correlated with macrophage M2 (Figure 7C). CA2 expression was significantly negatively correlated with macrophages M0 (Figure 7C).

### Expression levels of UC-related prognostic genes

**Figure 8:**
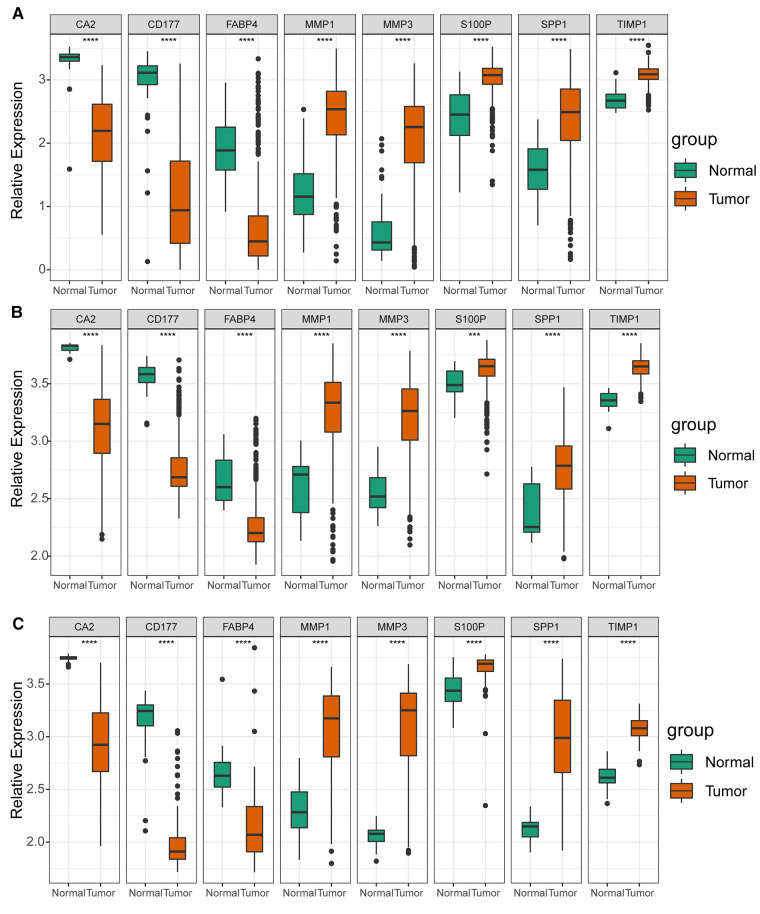
Expression levels of UC-related prognostic genes in three datasets. Expression levels of risk model genes in (**A**) TCGA-CRC, (**B**) GSE39582, and (**C**) GSE44076 datasets. **** *p* < 0.0001.

**Figure 9:**
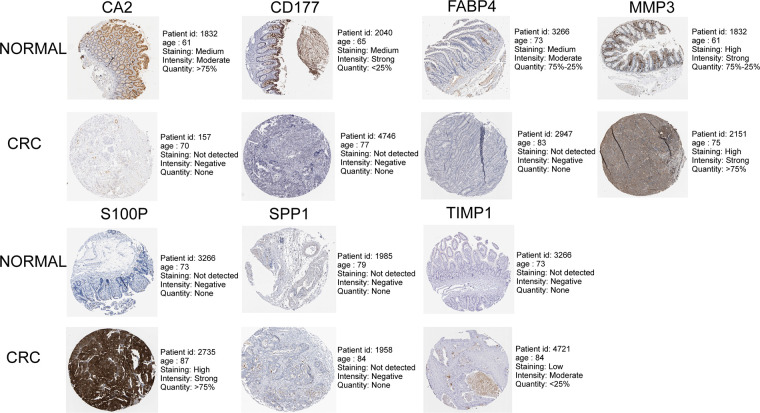
Immunohistochemistry images of UC-related prognostic genes obtained from the human protein atlas (HPA) database.

**Figure 10:**
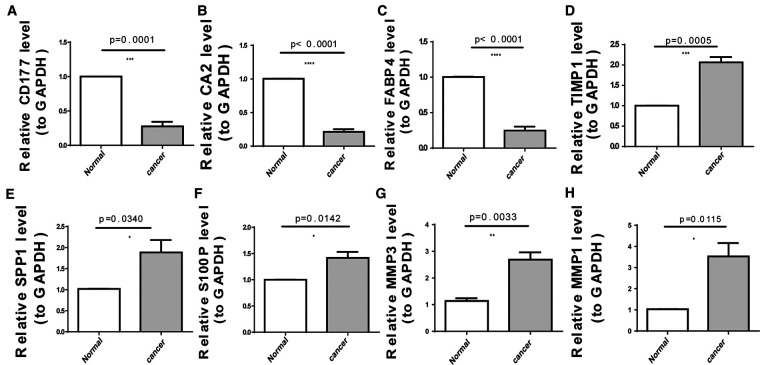
Expression levels of eight UC-related prognostic genes in clinical tissues determined using quantitative reverse transcription-polymerase chain reaction (qRT-PCR). (**A**) *CD177* (**B**) carbonic anhydrase 2 (*CA2*), (**C**) fatty acid-binding protein 4 (*FABP4*), (**D**) tissue inhibitor of metalloproteinase-1 (*TIMP1*), (**E**) secreted phosphoprotein 1 (*SPP1*), (**F**) S100 calcium-binding protein *P* (*S100P*), (**G**) matrix metallopeptidase (*MMP*)-*3*, and (**H**) *MMP1*. *p < 0.05, **p < 0.01, ***p < 0.001, ****p < 0.0001.

As illustrated in Figure 8A, CA2, CD177, and FABP4 levels were downregulated, while MMP1, MMP3, S100P, SPP1, and TIMP1 levels were elevated in CRC tissues compared to normal tissues in TCGA-CRC dataset. We further confirmed the same expression trend in the two external validation sets (GSE39582 and GSE44076) (Figures 8C–D). To further determine the changes in the expression of UC-related prognostic genes at the protein level, we obtained the corresponding immunohistochemistry images from HPA database. We did not detect immunohistochemical result of MMP1 CRC. As shown in Figure 9, we found that the protein expression levels of CA2, CD177, and FABP4 were reduced in CRC tissues than in normal tissues. SPP1 was largely unexpressed in normal and CRC tissues at the protein level. Moreover, protein expression levels of MMP3, S100P, and TIMP1 were increased in CRC tissues than in normal tissues. We verified the protein expression in clinical tissue samples *via* qRT-PCR. In agreement with the results of the public database data analysis, CA2, CD177, and FABP4 were expressed at low levels, while MMP1, MMP3, S100P, SPP1, and TIMP1 were highly expressed in clinical CRC samples compared to normal samples (Figure 10).

## Discussion

CRC is a molecularly heterogeneous malignancy with limited therapeutic strategies for patients with advanced disease. The molecular features of CRC are closely related to its prognosis, and there are no reliable prognostic biomarkers for risk prediction in clinical practice. Therefore, the identification of key biomarkers and therapeutic targets affecting prognosis is essential for improving the clinical outcomes in patients with CRC. As an important factor involved in cellular metabolic reprogramming, UC is involved in the genesis and development of various tumors, including CRC (20), revealing its potential as a prognostic biomarker. For the first time, we constructed and externally validated a novel prognostic model that integrated eight genes associated with UC. The genes constituting the prognostic model were *TIMP1*, *FABP4*, *MMP3*, *MMP1*, *CD177*, *CA2*, *S100P*, and *SPP1* (Table 4).

TIMP1 has been shown to promote the progression of multiple tumors (21–24). Studies in CRC have indicated that upregulation of TIMP1 was associated with poor prognosis and confirmed that TIMP1 can promote tumor cell proliferation and metastasis through the FAK/Akt signaling pathway (22, 25). FABP4 is a mediator of lipid metabolism in adipocytes and can provide fatty acids to tumor cells (26). FABP4 has been found to promote the progression of ovarian cancer, cervical cancer, breast cancer, prostate cancer cell carcinoma, and oral squamous cell carcinoma (26–30). In colorectal cancer, high expression of FABP4 has been found to be closely related to tumor recurrence (31). However, one study showed that FABP4 can inhibit the proliferation and metastasis of CRC cells (32). In this study, high expression of FABP4 was associated with a poor prognosis for CRC patients, so the mechanism of FABP4 in CRC needs further study. The bone bridge protein, SPP1, is a key ECM protein involved in tumor progression and metastasis (33) that is highly expressed in non-small cell lung cancer tissues (34), significantly upregulated in glioma and hepatocellular carcinoma cell lines, and *associated* with poor prognosis (35, 36). SPP1 expression is upregulated in CRC tissues and is associated with the short OS of patients (37), which is consistent with our experimental and predictive modeling results. MMP3 is a member of the metalloproteinase family along with MMP1, which affects tissue integrity by degrading ECM components (21). MMP3 is a member of the metalloproteinase family along with MMP1, which affects tissue integrity by degrading ECM components (21). Studies have shown that MMP3 can promote the metastasis of CRC (38), melanoma (39), and breast cancer (40). However, studies in CRC also found that the expression of MMP3 in patients without metastasis was significantly higher than that in patients with distant metastasis (41, 42). Our study also found a higher expression of MMP3 in low-risk patients. It is possible that MMP3 triggers tumor metastasis and the expression of MMP3 decreases after the tumor metastasizes. Previous studies have shown that MMP1 has potential as a diagnostic and prognostic marker for CRC (43, 44). Several studies have also confirmed that in CRC, the expression of MMP1 is lower in metastatic CRC than in primary CRC (45, 46). It indicates that MMP1 may have a similar role to MMP3, acting only in the initial stage of tumor metastasis and appearing to be less important once metastasis occurs. Therefore, the specific mechanism of action of MMP3 and MMP1 in colorectal cancer needs to be further studied. CD177 is a glycosylphosphatidylinositol-linked cell surface protein that is heterogeneously expressed by neutrophils, and its expression is associated with good prognosis in breast, prostate, cervical, and lung cancers (47). Studies have shown that CD177^+^ neutrophils inhibit epithelial tumorigenesis and serve as independent predictors of prognosis in patients with CRC (48). CA2 is a factor that inhibits metastasis and EMT and is associated with good OS in patients with hepatocellular carcinoma (49). CA2 is lowly expressed in ulcerative colitis and CRC tissues (50), and low CA2 expression is associated with a better prognosis. S100P is highly expressed in various solid tumors and associated with poor prognosis in CRC (51, 52), breast cancer (53), pancreatic cancer (54), cholangiocarcinoma (55) lung cancer (56), and ovarian cancer (57). Our qRT-PCR results also indicated that S100P was highly expressed in cancer tissues.

Our results showed that the risk score of the eight DEUCRGs is an independent prognostic marker for CRC patient survival. The survival rate of patients with CRC in the high-risk group was significantly lower than that in the low-risk group. The prognosis of the two risk groups differed in subgroups based on age, sex, clinical stage, T-stage, lymph node metastasis, distant metastasis, and race (Figure 4). Univariate and multivariate Cox regression analyses were used to further investigate the independent prognostic value of the clinicopathological characteristics and risk score, and the results showed that risk score, age, and T stage were significant independent prognostic factors for CRC (Figures 5A, B). An individualized prognostic prediction model was then constructed using a nomogram to quantify the individual risk in the clinical setting by integrating multiple risk factors, including independent prognostic factors, and calibration curves showed a high degree of agreement between the actual and predicted OS rates. Based on the above results, we suggest that our constructed prognostic risk score model is a valid prognostic indicator for patients with CRC.

GO and KEGG enrichment analyses of DEGs in different risk groups were used to determine the roles of UC-related biological processes and classical signaling pathways in different risk groups. As predicted, the results showed significant enrichment of genes in biological processes, such as muscle contraction, antimicrobial humoral response, and muscle system processes (Figure 6C). Changes in ECM components contribute to cancer progression, promote tumor-associated angiogenesis and inflammation, and affect the tumor microenvironment (58). Immune-related signaling pathways and functions, such as the humoral immune response and IL-17 signaling pathway, wernie also sigficantly enriched (Figure 6D). One study showed that the UC is associated with immunity (59). Single-sample GSEA scores showed significant differences in immune cell infiltration scores between the two risk groups. The proportions of B memory cells, T regulatory cells, monocytes, and macrophages M0 and M2 were elevated in the high-risk group, while the proportions of CD4 memory resting and activated T cells, plasma cells, activated dendritic cells, and neutrophils were elevated in the low-risk group (Figure 7B). Different levels of immune cell infiltration exist in different risk groups, and these differences may be important factors affecting the prognosis and treatment response of patients. These results suggest that targeting UC-related genes can potentially improve the immune status of CRC or promote immunotherapy in CRC; however, further studies are needed to confirm this.

Li et al. constructed a prognostic signature that included 25 long non-coding RNAs, but models incorporating too many variables were difficult to implement, limiting their clinical application (60). In contrast, the predictive models that we constructed may be easier to use in clinical practice. However, this study also has some limitations. Firstly, the molecular mechanisms of how urea cycle-associated genes in prognostic models affect the biological behaviour of colorectal cancer cells require further experimental validation. In addition, although our prognostic model was confirmed using an external dataset, further well-designed prospective studies are needed to validate these findings. In future studies, we aim to focus on the roles of these UCRGs in the prognosis of patients with CRC.

## Conclusions

In conclusion, we developed and validated a novel prognostic model based on the characteristics of eight UC-related genes in CRC for the first time, which will be of great significance in identifying prognostic molecular biomarkers, clinical prognosis prediction, and treatment strategy decisions for patients with CRC.

## Data Availability

The datasets presented in this study can be found in online repositories. The names of the repository/repositories and accession number(s) can be found in the article/**Supplementary Material**.
