# Supplementary material for: A novel prognostic model based on urea cycle-related gene signature for colorectal cancer
Source: Front Surg. 2022 Oct 21;9:1027655. doi: 10.3389/fsurg.2022.1027655 (PMC9633963; doi:10.3389/fsurg.2022.1027655)
Supplement: Supplementary file 2 [file Table2.docx]

**Table 4 The sequences of primers for qPCR**

| **primer** | **sequence** |
| --- | --- |
| FABP4 For | ACTGGGCCAGGAATTTGACG |
| FABP4 Rev | CTCGTGGAAGTGACGCCTT |
| TIMP1 For | TGTTGTTGCTGTGGCTGATAG |
| TIMP1 Rev | TCTGGTTGACTTCTGGTGTCC |
| SPP1 For | GGCTAAACCCTGACCCATCTC |
| SPP1 Rev | ATGGCTTTCGTTGGACTTACTTG |
| S100P For | CAAGGTGCTGATGGAGAAGGAG |
| S100P Rev | TTGTGACAGGCAGACGTGATTG |
| MMP3 For | ATGCTGTTGATTCTGCTGTTGAG |
| MMP3 Rev | ATTGGTCCCTGTTGTATCCTTTG |
| MMP1 For | CTCTGGAGTAATGTCACACCTCT |
| MMP1 Rev | TGTTGGTCCACCTTTCATCTTC |
| CD177 For | TGCCCAGTCTGCTTGTCTATG |
| CD177 Rev | GTGGTCCAATCAGTGGGTTCT |
| CA2 For | GAATGTGTGACCTGGATTGTGC |
| CA2 Rev | TTTGCCTGTTCTTCAGTGGCTG |
| GAPDH For | GGAAGGTGAAGGTCGGAGT |
| GAPDH Rev | TGAGGTCAATGAAGGGGTC |
