# Supplementary figures and images for: A novel prognostic model based on urea cycle-related gene signature for colorectal cancer
Source: Front Surg. 2022 Oct 21;9:1027655. doi: 10.3389/fsurg.2022.1027655 (PMC9633963; doi:10.3389/fsurg.2022.1027655)

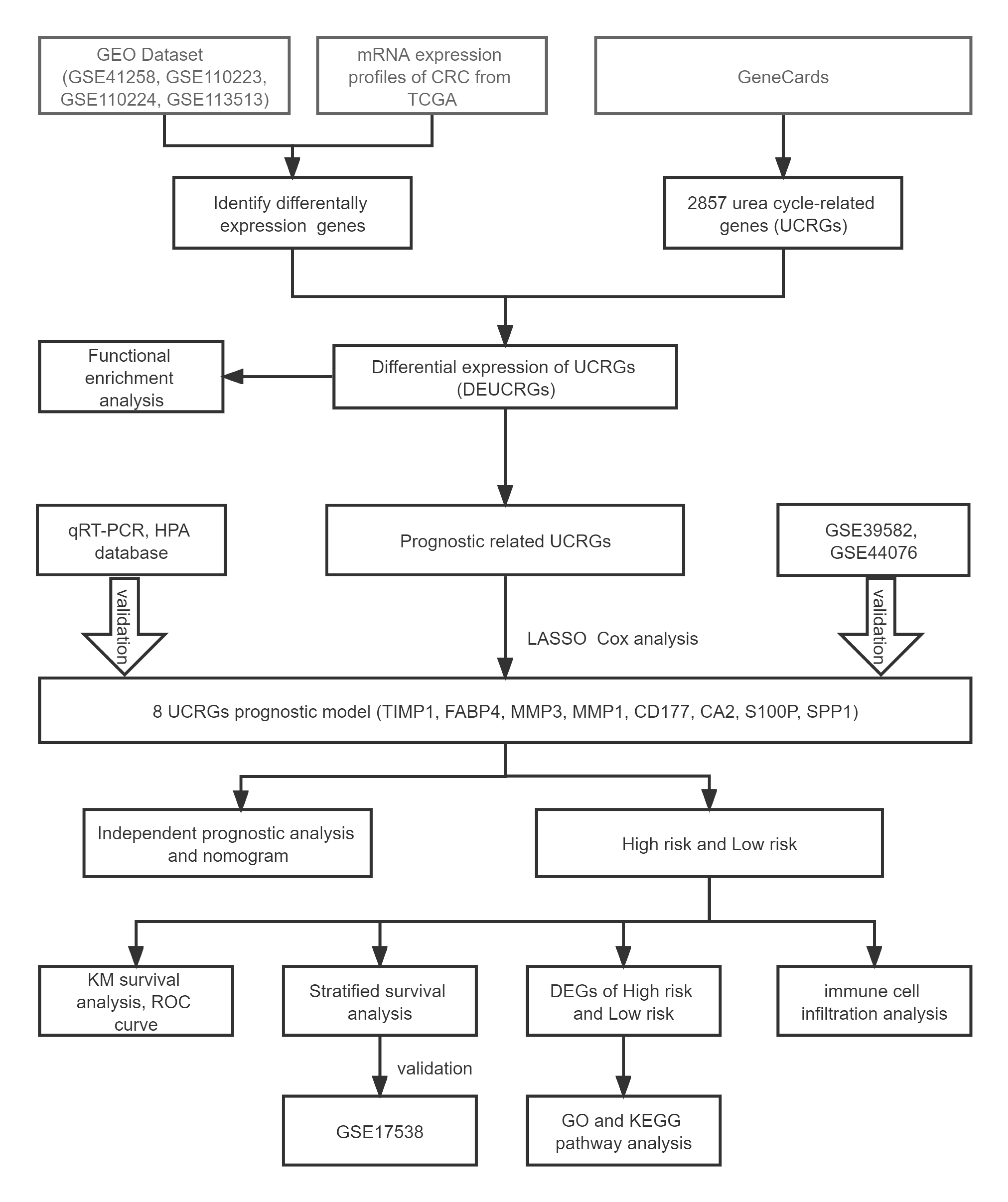

Supplement: Supplementary file 7 [file Image1.tif]

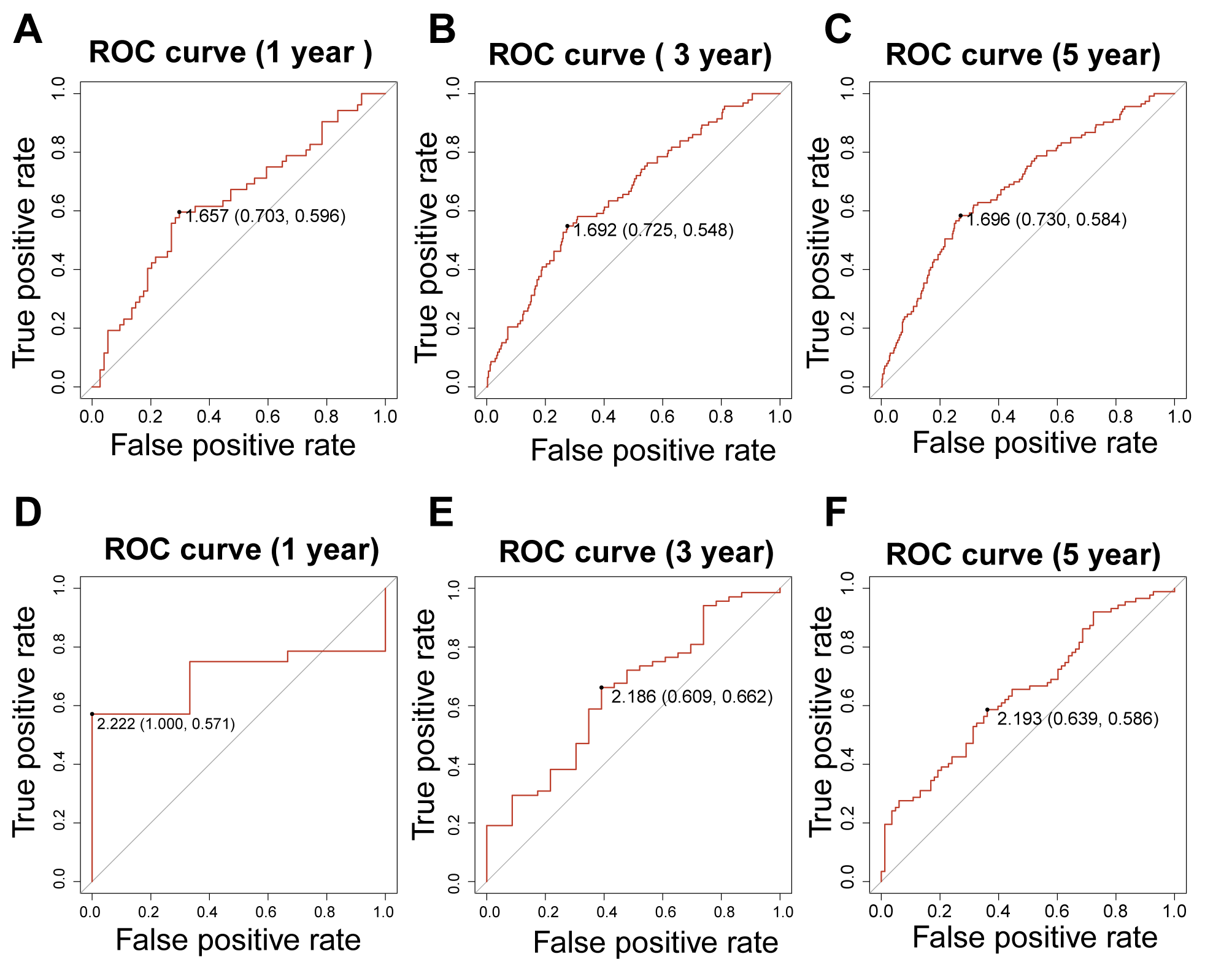

Supplement: Supplementary file 8 [file Image2.tif]
